# Commentary: Anthropometric Indicators as a Tool for Diagnosis of Obesity and Other Health Risk Factors: A Literature Review

**DOI:** 10.3389/fpsyg.2021.750613

**Published:** 2021-09-16

**Authors:** Hanen Samouda

**Affiliations:** Population Health Department, Luxembourg Institute of Health, Strassen, Luxembourg

**Keywords:** obesity, overweight, visceral fat, waist circumference, anthropometry, DEXA, MRI, CT-scan

## Introduction

We read with great interest the recently published article ≪ Anthropometric Indicators as a Tool for Diagnosis of Obesity and Other Health Risk Factors: A Literature Review ≫ (Piqueras et al., 2021). We felicitate the Authors as this a comprehensive literature review of the tools currently available to assess or diagnose obesity and potential related health issues.

Obesity is a chronic multifactorial disease and a global public health challenge, due to its high prevalence worldwide (World Health Organization, 2020) and the potential associated comorbidities, in particular in the presence of metabolically unhealthy obesity (MUO) (Samouda et al., 2019).

Body mass index (BMI) has widely been used to assess body fat accumulation characterizing obesity (WHO, 2000). However, BMI does not distinguish between fat mass and fat free mass, and therefore constitutes a poor diagnosis tool of obesity (Garn et al., 1986; Frankenfield et al., 2001; Adab et al., 2018). Yet, in order to manage a disease, we need first to diagnose it. Obesity diagnosis requires to have available easy to use and accurate tools to measure total and regional body fat storage, in particular visceral adiposity, major risk factor of developing obesity related comorbidities (Nicklas et al., 2006; Van Gaal et al., 2006; Cereda et al., 2007; Anan et al., 2010; Fontes-Carvalho et al., 2014; Yu et al., 2015; Brown et al., 2017, 2018; Han et al., 2017; Dan Lantsman et al., 2018; Kuritzkes et al., 2018; Magro et al., 2018; Tian et al., 2018).

## Additional Assessment Methods of Obesity

Dual-Energy-X-ray-Absorptiometry (DEXA) is the reference method for body composition assessment, providing a gold standard measurement of fat mass at both total and regional levels. Computed tomography scan (CT-scan) provide a reproducible and accurate measurement of visceral adipose tissue (VAT). Nevertheless, DEXA and CT-scan are considered as prohibitive techniques owing to the limited accessibility for the machines, the high cost of the imaging examination and the radiation exposure delivered by the multi-slice CT-scan protocols (Kvist et al., 1988; Jensen et al., 1993; Pritchard et al., 1993; Heymsfield et al., 1995).

Piqueras et al. (2021) reported 17 indices or health indicators to assess obesity using non-invasive and low-cost anthropometric measurements, which constitute a good alternative to expensive and less accessible biomedical imaging.

Regarding the anthropometric assessment of visceral adipose tissue, the authors stated that ≪ The model proposed by Samouda et al. (2013) correlates Visceral Adipose Tissue Area with the waist circumference and proximal thigh circumferences, BMI, and age, for adult men and women ≫ (Piqueras et al., 2021).

## Health Issues Associated With Obesity. Relationship to the Anthropometric Assessment Tools

Independently of the general fat mass accumulation, VAT depot has been highlighted as a major risk factor for developing several metabolic, cardiovascular, autoimmune, neurodegenerative, and oncological diseases, as well as an increased risk for early mortality (Van Gaal et al., 2006; Cereda et al., 2007; Fontes-Carvalho et al., 2014; Yu et al., 2015; Brown et al., 2017, 2018; Dan Lantsman et al., 2018; Kuritzkes et al., 2018; Magro et al., 2018). The Authors reported a significant relationship between the identified anthropometric tools assessing total and regional adiposity and several health issues, including glucose dysregulation, insulin resistance, metabolic syndrome, cardiometabolic dysregulation, type 2 diabetes, hypertension, cardiovascular diseases, cancer, as well as a higher risk of early cardiovascular and all-cause mortality (Piqueras et al., 2021).

## Discussion

Following these statements, we would like to clarify that our previous work related to the ≪ Innovative anthropometric model to predict visceral adipose tissue without resort to CT-Scan or DXA ≫ (Samouda et al., 2013) did not aim to simply correlate the visceral adipose tissue with the waist circumference (Waist C), proximal thigh circumference (Proximal Thigh C), body mass index (BMI) and age.

In order to develop our tools, multiple linear regressions with an empirical selection of the variables were developed by no controlled stepwise regressions (Samouda et al., 2013). The anthropometric VAT model we developed assumed that if we subtract the most correlated anthropometric measurement with subcutaneous abdominal adipose tissue (SAAT) from the most correlated anthropometric measurement with total abdominal adipose tissue (TAAT) and VAT as assessed by CT-Scan, we obtain the most accurate prediction of VAT by anthropometry. In our tools, Proximal Thigh C was the most correlated (R Pearson) anthropometric measurement with SAAT as assessed by CT-Scan, respectively Waist C was the most correlated anthropometric measurement with TAAT and VAT (Samouda et al., 2013).

These tools have been developed in 253 patients from south Europe, aged between 18 and 78 years (BMI: 16 to 53 kg/m^2^):

- “In Women:
VAT = (2.15 × Waist C) – (3.63 × Proximal Thigh C) + (1.46 × Age) + (6.22 × BMI) - 92.713
- In Men:
VAT = (6 × Waist C) – (4.41 × proximal thigh C) + (1.19 × Age) - 213.65” (Samouda et al., 2013).

The Authors also stated that there was no specific cut-offs for VAT accumulation (Piqueras et al., 2021). Actually, the cut-off of 130 cm^2^ of VAT has previously been defined by Hunter et al. (1994) as associated with an increased risk of hypertension and dyslipidemia. The ability of our selected anthropometric tools for the diagnosis of a VAT excess ≥ 130 cm^2^ was considerably high: sensitivity (97.7% in women, 100% in men), specificity (75% in men, 85.7% in women), positive predictive values (91.3% in women, 90.9% in men), and negative predictive values (96% in women, 100% in men) (Samouda et al., 2013).

In addition, the VAT anthropometric tools we developed have been validated as being the most accurate predictors of cardiometabolic abnormalities, cancer and early mortality (cardiovascular, cancer, and all causes), compared to BMI and Waist C, when biomedical imaging are not available. This validation was conducted in 10.624 participants of European descent to the National Health and Nutrition Examination Survey followed for 20 years (Brown et al., 2017, 2018). Similar findings were observed in a population-based study in North Europe (Ruiz-Castell et al., 2021).

Finally, we emphasize the potentialities offered by our innovative anthropometric method, which offer accurate and easy to use tools to predict VAT without resort to biomedical imaging.

## Author Contributions

HS drafted the Commentary.

## Conflict of Interest

The author declares that the research was conducted in the absence of any commercial or financial relationships that could be construed as a potential conflict of interest.

## Publisher's Note

All claims expressed in this article are solely those of the authors and do not necessarily represent those of their affiliated organizations, or those of the publisher, the editors and the reviewers. Any product that may be evaluated in this article, or claim that may be made by its manufacturer, is not guaranteed or endorsed by the publisher.
